# Relationship Between Programmed Death Ligand 1 Expression and Other Clinicopathological Features in a Large Cohort of Gastric Cancer Patients

**DOI:** 10.3389/fimmu.2022.783695

**Published:** 2022-03-25

**Authors:** Xinhua Chen, Huimin Zhang, Minghao Wang, Hao Liu, Yanfeng Hu, Tian Lin, Hao Chen, Mingli Zhao, Tao Chen, Guoxin Li, Jiang Yu, Liying Zhao

**Affiliations:** ^1^ Department of General Surgery and Guangdong Provincial Key Laboratory of Precision Medicine for Gastrointestinal Tumor, Nanfang Hospital, The First School of Clinical Medicine, Southern Medical University, Guangzhou, China; ^2^ The First Clinical Medical School, Southern Medical University, Guangzhou, China

**Keywords:** gastric cancer, PD-L1, CPS, Ki-67, biopsy, pathology

## Abstract

**Background:**

Antibodies against programmed death 1 (PD-1) and its ligand, programmed death-ligand 1 (PD-L1) have recently shown promising results in gastric cancer (GC). However, clinicians still lack predictive biomarkers for the efficacy of anti-PD-1 therapy; thus, we investigated the expression of PD-L1 in GC and further assessed its clinical relevance with other clinicopathological features.

**Methods:**

We retrospectively collected clinical data on 968 consecutive GC cases from Nanfang Hospital between November 2018 and August 2021. Discrepancy in the combined positive score (CPS) of PD-L1 protein expression between gastric mucosa biopsy and postoperative pathology were investigated. Correlations between CPS and clinicopathological parameters were determined using chi-squared test, multiple logistic aggression analysis, and linear regression analysis.

**Results:**

Among the 968 consecutive GC patients, 199 who did not receive preoperative chemotherapy or immunotherapy were tested for CPS both in gastric mucosa biopsy and postoperative pathology, and the results showed that the CPS of gastric mucosa biopsy was significantly lower than that of postoperative pathology [mean ± SD: 5.5 ± 9.4 vs. 13.3 ± 17.4; M(IQR): 2(5) vs. 5(12), p<0.001)]. 62.3% of patients (579/930) had CPS≥ 1, 49.2% of patients (458/930) had CPS≥5, and 33.3% of patients (310/930) had CPS≥10. Mismatch repair deficiency (dMMR) status was seen in 6.1% of patients (56 of 919). Positive Epstein–Barr virus (EBV) status was detected in 4.4% of patients (38 of 854). The patients with CPS≥1/CPS≥5/CPS≥10 were significantly independently correlated with age, Lauren classification, Ki-67 index, and EBV status. According to linear regression analysis, PD-L1 expression was correlated with age (p<0.001), Ki-67 index (p<0.001), EBV (p<0.001), and Lauren classification (p=0.002).

**Conclusions:**

Our results confirmed that PD-L1 expression has Intratumoral heterogeneity in GC. Furthermore, the variables of age, Ki-67 index, and Lauren classification, which are common and accessible in most hospitals, are worth exploring as potential biomarkers for anti-PD-1 therapy in GC.

## Introduction

Blocking immune checkpoint molecules with monoclonal antibodies has recently emerged as a promising strategy for treating some malignancies (1–5). Currently, The immune regulatory programmed death-1 (PD-1)/programmed death-ligand 1 (PD-L1) axis has been used as a checkpoint target for immunotherapy (6). Anti-PD-1/PD-L1 therapy has also shown promising antitumor activity in gastric cancer (GC) (4, 7, 8). Although there are no established biomarkers for anti-PD-1/PD-L1 antibodies, PD-L1 expression, Epstein–Barr virus (EBV) status, and DNA mismatch repair (MMR) status have been proposed as predictive biomarkers for anti-PD-1 response (9, 10). The CheckMate-032 trial showed a greater association of PD-L1 expression by combined positive score (CPS) with anti-PD1 therapy efficacy (11). However, the percentages of CPS ≥1, ≥5, and ≥10 were 32%, 10%, and 8%, respectively. Additionally, all CPSs for GC are decided by tests on tissue from gastric mucosa biopsy. Whether the CPS characteristics of resectable GC are different from the CheckMate-032 trial remains unknown. There are still no predictive biomarkers for the clinical efficacy of anti-PD-1 therapy. Currently, the main indication for anti-PD-1 therapy in GC is positive PD-L1 expression. However, since immune therapy have reshaped the paradigm of cancer therapy and progress rapidly in GC treatment in reccent year, the immunohistochemical (IHC) for immune therapy has not yet been updated or popularized in most hospital. In China, the area with the highest GC incidence, most hospitals cannot adequately assess PD-L1 expression for GC patients. This phenomenon inhibits the use of anti-PD-1 therapy in GC. In addition, associations between PD-L1 expression and other clinicopathological features, which urgently need to be better understood, remain unclear. Thus, we investigated PD-L1 expression in GC and discrepancy in CPS between gastric mucosa biopsy and postoperative pathology. Finally, we assessed the clinical associations between PD-L1 expression and other clinicopathological features that are more accessible in Chinese hospitals.

## Methods

### Tumor Specimens and Clinical Data Collection

**Figure 1:**
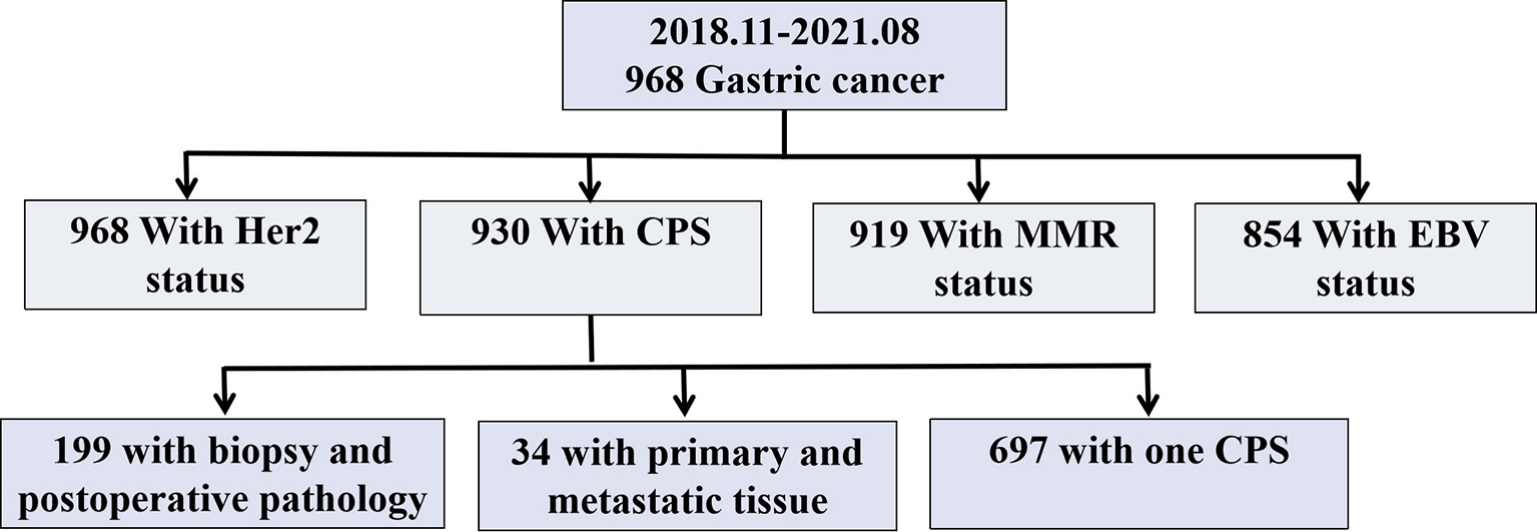
Study Chart. Her 2, Human epidermal growth factor receptor 2; CPS, Combined Positive Score; MMR, Mismatch repair; EBV, Epstein-Barr virus.

The study was approved by the Clinical Research Ethics Committee of Nanfang Hospital, Southern Medical University. Written informed consent was obtained from all participants included in the study. In total, 968 GC cases were collected from the files of the Department of Pathology, Nanfang Hospital (**Figure 1**). All cases were reviewed by two pathologists, and histological diagnoses were confirmed without discrepancy. Clinical characteristics including age, body mass index (BMI), sex, diabetes (12), tumor location (13), histology, Lauren classification, grade, Tumor size, T stage (14), N stage (15, 16), M stage (14), Ki-67 index, S-100, CD-31, D240, EBV, MMR, and, HER2 statuses, and routine blood indicators [white blood cell (WBC), mononuclear cell (MONO), eosinophilic granulocyte (EOS), neutrophil (NEU), lymphocyte (LYM), and platelet (PLT) counts, the neutrophil/lymphocyte ratio (NLR), and ABO blood group] were obtained from medical records, pathology reports, discharge summaries, and extracted from the prospective database. All procedures followed were in accordance with the ethical standards of the responsible committee on human experimentation (institutional and national) and with the Helsinki Declaration of 1964 and later versions. The data collection protocol was approved by the Ethics Committee of Nanfang Hospital, Southern Medical University. Informed consent to be included in the study, was obtained from all patients.

### Immunohistochemical (IHC) Staining and Evaluation

**Figure 2:**
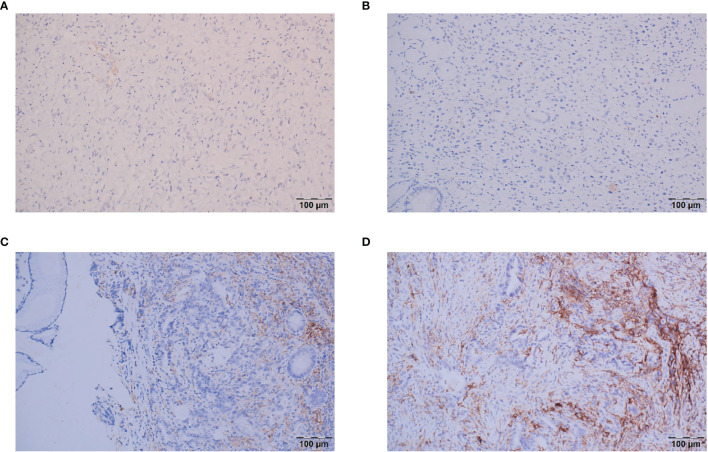
Representative images of the different CPS categories. **(A)** CPS<1; **(B)** CPS=1; **(C)** CPS=5; **(D)** CPS≥10.

IHC was performed on 4-μm-thick tissue sections using an automated IHC stainer (Ventana, Tucson, AZ, USA). The assessment of PD-L1 protein expression in GC is a qualitative immunohistochemical assay that uses anti-PD-L1 antibodies(Dako, 22C3) to detect PD-L1 protein in formalin-fixed, paraffin-embedded (FFPE) tissues from gastric adenocarcinomas. A minimum of 100 tumor cells must be present in the PD-L1-stained slide for the specimen to be considered adequate for PD-L1 evaluation. A specimen is considered to have PD-L1 expression if CPS≥1. CPS is the total number of positively stained PD-L1 cells (i.e., tumor cells, lymphocytes, and macrophages) divided by the total number of viable tumor cells, multiplied by 100%. For the patients with CPS in both biopsy and postoperative samples, the final CPS was decided by the higher scores. And the CPS categories in this study were classified as CPS<1, CPS≥1, CPS≥5 and CPS≥10 (**Figure 2**).

MMR status was assessed by IHC using monoclonal antibodies for anti-mutL homolog 1 (MLH1, Abnova, M1), anti-mutS homolog 2 (MSH2, BD Bioscience, G219-1129), anti-postmeiotic segregation increased 2 (PMS2,BD Bioscience, A16-4), and anti-mutS homolog 6 (MSH6, Abcam, SP93). Tumors lacking either MLH1, MSH2, PMS2, or MSH6 expression were considered MMR deficient (dMMR), whereas tumors that maintained expression of MLH1, MSH2, PMS2, and MSH6 were considered MMR proficient (pMMR). HER2 expression, which was monitoring of IHC stains using monoclonal antibodies (Ventana, 4B5), was graded using a score scale of 0 to 3 (17). Chromogenic *in situ* hybridization for EBV-encoded RNA (EBER) using fluorescein-labeled oligonucleotide probes (ZSGB-BIO, ISH-7001) was performed to assess EBV status (18).

### Statistical Analysis

Data are presented as mean ± standard deviation for continuous variable (for those with non-normal distribution, median and interquartile range (IQR) are shown) and as number (%) for categorical variables. The Student’s t-test/paired t-test and Mann–Whitney U test were used to compare continuous variables, and the χ^2^ test and Fisher’s exact test were used to compare categorical variables, as appropriate.

Risk factors for PD-L1 expression were evaluated by uni- and multi-variate analyses using logistic regression models. Based on multivariate logistic regression analysis, the linear regression analysis was performed to demonstrate the linear correlation of CPS and selected clinicopathological characteristics. Statistically significant variables (p<0.05) in univariate analysis were entered into the multivariable model and were analyzed by using an “Enter” method. Enter is the mandatory method, which means that all the variables we choose are analysed in the model. A two-tailed p-value <0.05 was considered statistically significant. SPSS version 25.0 (IBM Corp., Armonk, NY, USA) was used to analyze all data.

## Results

### PD-L1 Expression and MMR and EBV Status in GC Patients

**Figure 3:**
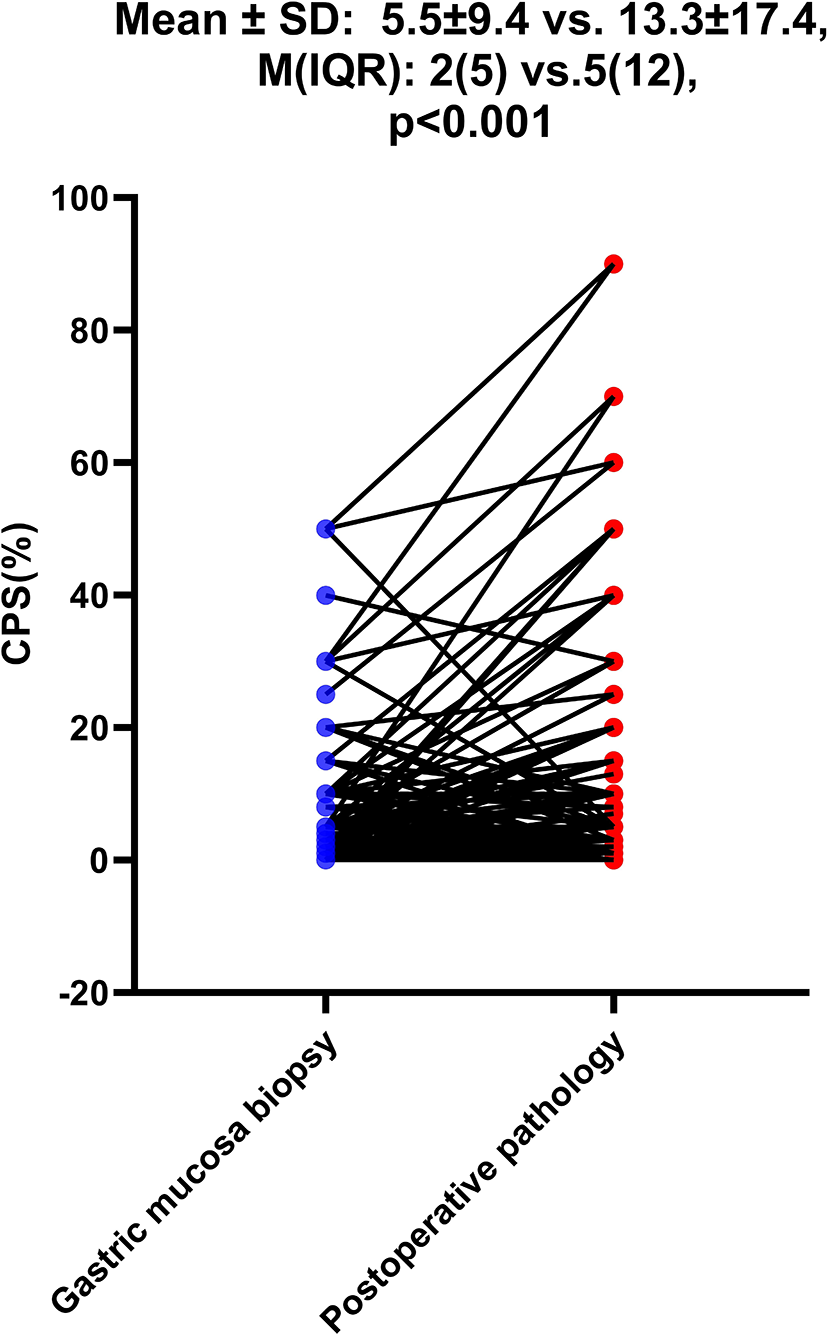
The difference of CPS between gastric mucosa biopsy and postoperative pathology. The Student’s paired t-test was used to compare them.

**Figure 4:**
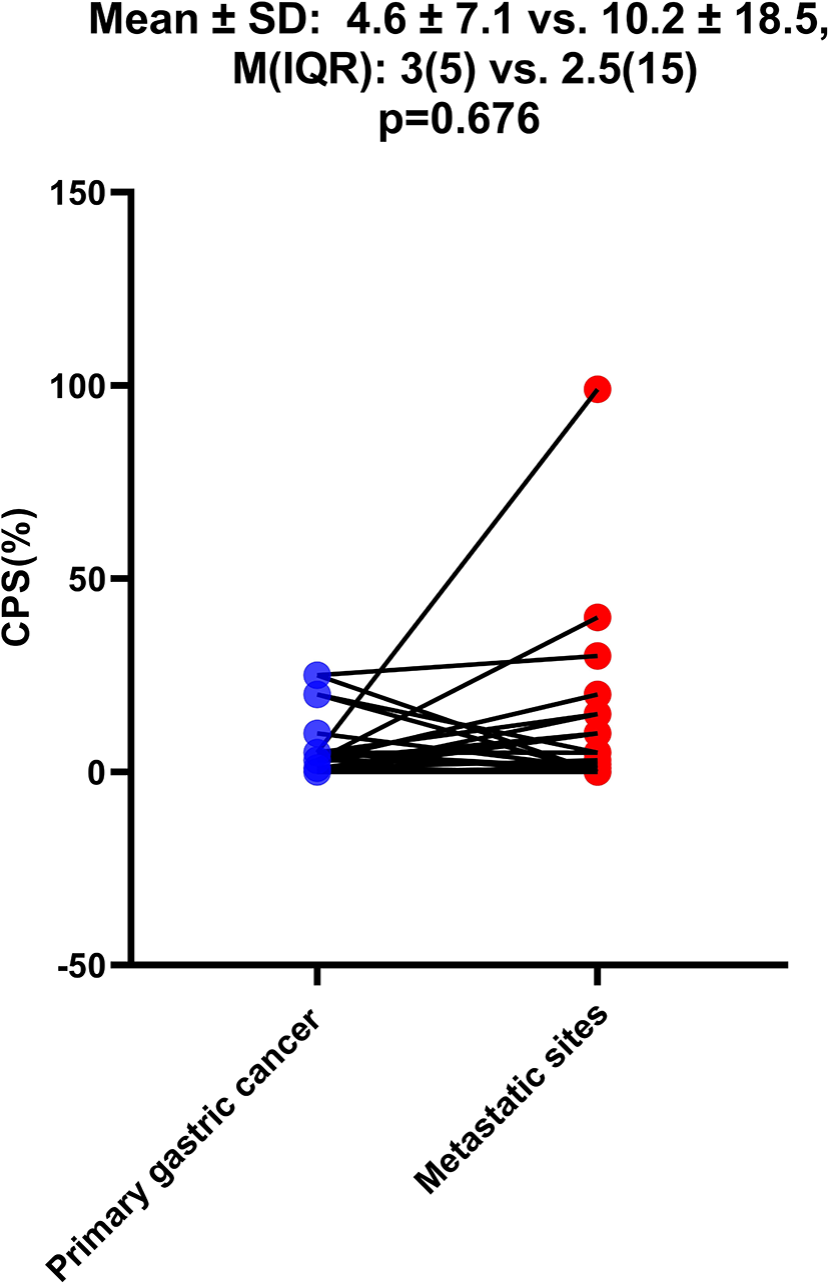
The difference of CPS between primary gastric cancer and metastatic sites. The Student’s paired t-test was used to compare them.

As shown in **Table 1**, among the 968 consecutive GC patients, 199 did not undergo preoperative chemotherapy or immunotherapy and had CPS tested both in gastric mucosa biopsy and postoperative pathology. From these different tissue samples, the results showed that the CPS of gastric mucosa biopsy was significantly underestimated compared with that in postoperative pathology (mean ± SD: 5.5 ± 9.4 vs. 13.3 ± 17.4; M(IQR): 2(5) vs. 5(12), p<0.001) (**Figure 3**). The CPS of primary gastric cancer was lower than those of metastatic sites, but the difference was not significant (mean ± SD: 4.6 ± 7.1 vs. 10.2 ± 18.5; M(IQR): 3(5) vs. 2.5(15), p=0.676) (**Figure 4**). As shown in **Table 2**, 62.3% of patients (579/930) expressed PD-L1(CPS≥ 1), 49.2% of patients (458/930) showed CPS≥ 5, and 33.3% of patients (310/930) showed CPS≥10. dMMR status was observed in 6.1% of patients (56/919). EBV positive status was detected in 4.4% of patients(38/854) while HER-2 ++/+++ status was observed in 12.0% of patients(116/968).

**Table 1:** The CPS of gastric cancer test by tissue from gastric mucosa biopsy and postoperative pathology, primary gastric cancer and metastatic sites.

	Mean ± SD	M(IQR)	p
CPS of initial diagnosed GC			<0.001
Gastric mucosa biopsy	5.5 ± 9.4	2 (5)	
Postoperative pathology	13.3 ± 17.4	5 (12)	
CPS of primary gastric cancer and metastasis site			0.676
Primary gastric cancer	4.6 ± 7.1	3 (5)	
Metastasis site	10.2 ± 18.5	2.5 (15)	

CPS, combined positive score.

**Table 2:** PD-L1 Expression (CPS), MMR and EBV Status and HER-2 Status in Gastric Cancer.

Variables	Cases [n(%)]
CPS ≥1	
No	351 (37.7)
Yes	579 (62.3)
CPS ≥5	
No	472 (50.8)
Yes	458 (49.2)
CPS ≥10	
No	620 ( 66.7)
Yes	310 (33.3)
dMMR	
No	863 (93.9)
Yes	56 (6.1)
EBV positivity	
No	816 (95.6)
Yes	38 (4.4)
HER-2 :++ or +++	
No	852 (88.0)
Yes	116 (12.0)

CPS, combined positive score; EBV, The Epstein-Barr virus, MMR, Mismatch repair; dMMR, mismatch repair deficiency; HER-2, Human epidermal growth factor receptor 2.

The Higher level(2+/3+) of reactions for immunohistochemical HER-2 tests.

### Correlations Between PD-L1 Expression and Clinicopathological Characteristics

#### Clinicopathological Features Associated With PD-L1 Expression of CPS≥1

As shown in **Table 3**, PD-L1 expression of CPS≥1 was significantly associated with age (CPS≥1 vs. CPS<1: 58.4 ± 12.2 vs. 56.1 ± 12.8, p=0.008), Lauren classification (CPS≥1 vs. CPS<1: intestinal/diffuse/mix: 24.8%/58.9%/16.3% vs. 34.4%/55.3%/10.3%, p=0.005), tumor size≥5 cm (CPS≥1 vs. CPS<1: 32.5% vs. 25.6%, p=0.027), CD-31 positivity (CPS≥1 vs. CPS<1: 25.2% vs. 18.0%, p=0.022), EBV positivity (CPS≥1 vs. CPS<1: 6.1% vs. 1.6%, p=0.001), Ki-67 index (CPS≥1 vs. CPS<1: 60.3% ± 23.8% vs. 52.6% ± 23.9%, p<0.001), and EOS count [CPS≥1 vs. CPS<1: 1.8 ± 1.6 (×10^8^/L) vs. 1.6 ± 1.2 (×10^8^/L), p=0.042]. In contrast, PD-L1 expression of CPS≥1 was not associated with BMI (p=0.968), sex (p=0.729), diabetes (p=0.338), tumor location (p=0.116), histology (p=0.691), grade (p=0.088), tumor depth (p=0.196), lymph node stage (p=0.482), metastasis stage (p=0.299), S-100 (p=0.340), D-240 (p=0.802), dMMR status (p=0.979), or HER-2 status (p=0.055). Hemocyte data including the counts of WBC (p=0.584), MONO (p=0.223), NEU (p=0.375), LYM (p=0.118), and PLT (p=0.056), the NLR (p=0.167), and ABO blood groups (p=0.705) were similar between the two groups. Multiple logistic regression analyses (**Table 4**) showed that age (hazard ratio [HR]: 1.022, 95% confidence interval [95%CI]: 1.008–1.036, p=0.001), Lauren classification (diffuse vs. intestinal: HR: 1.898, 95%CI: 1.301–2.752, p=0.001; mix vs. intestinal: HR: 2.052, 95%CI: 1.211–3.477, p=0.008), Ki-67 index (HR: 1.010, 95%CI: 1.003–1.017, p=0.003), and EBV status (positive vs. negative: HR: 3.318, 95%CI: 1.112–9.903) were independently associated with the frequency of CPS≥1.

**Table 3:** Clinicopathological features associated with PD-L1 expression of CPS ≥ 1.

Variables	CPS < 1	CPS≥1	Statistic	p-value
Age(mean±SD)	56.1 ± 12.8	58.4 ± 12.2	-2.638	0.008
BMI(mean±SD)	22.5 ± 2.9	22.4 ± 3.3	0.040	0.968
Sex[n(%)]			0.120	0.729
Male	224 (63.8)	376 (64.9)		
Female	127 (36.2)	203 (35.1)		
Diabetes[n(%)]			0.916	0.338
No	318 (90.6)	513 (88.6)		
Yes	33 (9.4)	66 (11.4)		
Location[n(%)]			4.303	0.116
Upper	76 (21.7)	123 (21.2)		
Middle	79 (22.5)	100 (17.3)		
Lower	196 (55.8)	356 (61.5)		
Histology[n(%)]			0.157	0.691
Sig	127 (36.2)	217 (37.5)		
Others	224 (63.8)	362 (62.5)		
Lauren classification[n(%)]			10.664	0.005
Intestinal	100 (34.4)	116 (24.8)		
Diffuse	161 (55.3)	275 (58.9)		
Mix	30 (10.3)	76 (16.3)		
Grade[n(%)]			6.543	0.088
G1	23 (6.6)	25 (4.3)		
G2	67 (19.1)	83 (14.3)		
G3	258 (73.5)	466 (80.5)		
G4	3 (0.9)	5 (9.9)		
Tumor size[n(%)]			4.862	0.027
<5cm	261 (74.4)	391 (67.5)		
≥5cm	90 (25.6)	188 (32.5)		
T stage [n(%)]			-1.294	0.196
T1a	49 (14.0)	70 (12.1)		
T1b	32 (9.1)	53 (9.2)		
T2	39 (11.1)	52 (9.0)		
T3	92 (26.2)	147 (25.4)		
T4a	95 (27.1)	183 (31.6)		
T4b	44 (12.5)	74 (12.8)		
N stage [n(%)]			-0.703	0.482
N0	138 (39.3)	207 (35.8)		
N1	38 (10.8)	62 (10.7)		
N2	42 (12.0)	91 (15.7)		
N3	133 (37.9)	219 (37.8)		
Metastasis [n(%)]			1.077	0.299
No	263 (74.9)	451 (77.9)		
Yes	88 (25.1)	128 (22.1)		
Ki-67(%)( mean±SD)	52.6 ± 23.9	60.3 ± 23.8	4.487	<0.001
S-100[n(%)]			0.912	0.340
Negative	129 (43.7)	185 (40.2)		
Positive	166 (56.3)	275 (59.8)		
CD-31[n(%)]			5.274	0.022
Negative	241 (82.0)	348 (74.8)		
Positive	53 (18.0)	117 (25.2)		
D-240[n(%)]			0.063	0.802
Negative	207 (70.2)	323 (69.3)		
Positive	88 (29.8)	143 (30.7)		
EBV[n(%)]			9.566	0.002
Negative	309 (98.4)	506 (93.9)		
Positive	5 (1.6)	33 (6.1)		
dMMR[n(%)]			0.001	0.979
No	324 (93.9)	536 (93.9)		
Yes	21 (6.1)	35 (6.1)		
Her2[n(%)]			-1.921	0.055
0	249 (70.9)	370 (63.9)		
+	60 (17.1)	137 (23.7)		
++	23 (6.6)	43 (7.4)		
+++	19 (5.4)	29 (5.0)		
WBC (x 10~9/L)(mean±SD)	6.0 ± 1.8	6.1 ± 2.1	-0.459	0.584
MONO(x 10~9/L)(mean±SD)	0.5 ± 0.2	0.5 ± 1.3	-1.219	0.223
EOS(x 10~8/L)(mean±SD)	1.6 ± 1.2	1.8 ± 1.6	-2.185	0.042
NEU(x 10~9/L)(mean±SD)	3.5 ± 1.6	3.6 ± 1.8	-0.911	0.375
LYM(x 10~9/L)(mean±SD)	1.8 ± 0.6	1.7 ± 0.7	1.564	0.118
NLR(mean±SD)	2.2 ± 1.7	2.4 ± 1.7	-1.382	0.167
PLT(mean±SD)	245.8 ± 79.6	256.8 ± 93.6	-1.914	0.056
ABO blood group[n(%)]			1.404	0.705
A	109 (31.2)	170 (29.6)		
B	72 (20.6)	136 (23.7)		
AB	29 (8.3)	51 (8.9)		
O	139 (39.8)	217 (37.8)		

CPS, combined positive score; BMI, body mass index; EGJ, esophagus-gastric junction; EBV, The Epstein-Barr virus; dMMR, mismatch repair deficiency; Her 2, Human epidermal growth factor receptor 2; WBC, white blood cell count; MONO, mononuclear cell count; EOS, eosinophilic granulocyte count; NEU, neutrophil count; LYM, lymphocyte count; NLR, neutrophil/lymphocyte ratio; PLT, the platelet count.

**Table 4:** The Multivariate Logistic Regression Analyses of the PD-L1 expression of CPS ≥ 1.

Variables	B	S.E.	P value	HR (95%CI)
Age	0.022	0.007	0.001	1.022 (1.008-1.036)
Lauren classification (Diffuse/Intestinal)	0.638	0.191	0.001	1.898 (1.301-2.752)
Lauren classification (Mix/Intestinal)	0.719	0.269	0.008	2.052 (1.211-3.477)
Size(≥5cm/<2cm)	0.066	0.200	0.743	1.068 (0.722-1.580)
Ki-67	0.010	0.003	0.003	1.010 (1.003-1.017)
CD31(+/-)	0.268	0.201	0.183	1.308 (0.881-1.940)
EBV(+/-)	1.199	0.558	0.032	3.318 (1.112-9.903)
EOS	0.703	0.568	0.216	2.017 (0.664-6.148)

CPS, combined positive score; EBV, The Epstein-Barr virus; EOS, eosinophilic granulocyte count; B, regression coefficient; S.E., standard error.

#### Clinicopathological Features Associated With PD-L1 Expression of CPS≥5

As shown in **Table S1**, PD-L1 expression of CPS≥5 was significantly related to age (CPS≥5 vs. CPS<5: 59.0 ± 12.0 vs. 56.1 ± 12.7, p<0.001), tumor size≥5 cm (CPS≥5 vs. CPS<5: 33.0% vs. 26.9%, p=0.043), Lauren classification (CPS≥5 vs. CPS<5: intestinal/diffuse/mix: 25.4%/57.8%/16.8% vs. 31.5%/57.3%/11.2%, p=0.033), CD-31 positivity (CPS≥5 vs. CPS<5: 27.5% vs. 17.5%, p=0.001), D240 positivity (CPS≥5 vs. CPS<5: 34.0% vs. 26.9%, p=0.034), EBV positivity (CPS≥5 vs. CPS<5: 7.3% vs. 1.6%, p<0.001), and the Ki-67 index (CPS≥5 vs. CPS<5: 61.3% ± 23.3% vs. 53.6% ± 24.3%, p<0.001). In contrast, there was no clear relationship with BMI (p=0.393), sex (p=0.839), diabetes (p=0.080), tumor location (p=0.287), histology (p=0.848), grade (p=0.112), tumor depth (p=0.930), lymph node stage (p=0.987), metastasis stage (p=0.077), S-100 (p=0.298), MMR status (p=0.228), or HER-2 status (p=0.394). Additionally, hemocyte data including the counts of WBC (p=0.807), MONO (p=0.132), EOS (p=0.077), NEU (p=0.882), LYM (p=0.059), and PLT (p=0.244), the NLR (p=0.228), and ABO blood groups (p=0.607) were similar between the two groups. Logistic regression analyses (**Table S2**) showed that age (HR: 1.029, 95%CI: 1.015–1.043, p<0.001), Lauren classification (diffuse vs. intestinal: HR: 1.579, 95%CI: 1.088–2.292, p=0.016; mix vs. intestinal: HR: 1.797, 95%CI: 1.077–2.977, p=0.025), Ki-67 index (HR: 1.011, 95%CI: 1.004–1.018, p=0.002), and EBV status (positive vs. negative: HR: 4.439, 95%CI: 1.620–12.160) were independently associated with the frequency of CPS≥5.

#### Clinicopathological Features Associated With PD-L1 Expression of CPS≥10

As shown in **Table 5**, PD-L1 expression of CPS≥10 was significantly related to age (CPS≥10 vs. CPS<10: 60.3 ± 11.3 vs. 56.1 ± 12.8, p<0.001), tumor size≥5 cm (CPS≥10 vs. CPS<10: 37.7% vs. 26.0%, p<0.001), Lauren classification (CPS≥10 vs. CPS<10: intestinal/diffuse/mix: 25.6%/56.1%/18.3% vs. 29.9%/58.2%/11.9%, p=0.049), CD-31 positivity (CPS≥10 vs. CPS<10: 28.0% vs. 19.7%, p=0.010), EBV positivity (CPS≥10 vs. CPS<10: 10.7% vs. 1.2%, p<0.001), dMMR status (CPS≥10 vs. CPS<10: 8.8% vs. 4.8%, p=0.015), and the Ki-67 index (CPS≥10 vs. CPS<10: 64.7% ± 22.9% vs. 53.7% ± 23.9%, p<0.001). In contrast, PD-L1 expression of CPS≥10 was not associated with BMI (p=0.131), sex (p=0.663), diabetes (p=0.652), tumor location (p=0.083), histology (p=0.093), grade (p=0.138), tumor depth (p=0.260), lymph node stage (p=0.694), metastasis stage (p=0.188), S-100 (p=0.888), D-240 (p=0.071), or HER-2 status (p=0.411). Additionally, hemocyte data including the counts of MONO (CPS≥10 vs. CPS<10: 0.6 ± 1.7 vs. 0.5 ± 0.2, p=0.022), EOS (CPS≥10 vs. CPS<10: 1.9 ± 1.8 vs. 1.7 ± 1.3, p=0.021), LYM (CPS≥10 vs. CPS<10: 1.7 ± 0.7 vs. 1.8 ± 0.7, p=0.001) and NLR (CPS≥10 vs. CPS<10: 2.5 ± 1.9 vs. 2.2 ± 1.5, p=0.005) were significantly different, while the counts of WBC (p=0.619), NEU (p=0.240), PLT (p=0.217), and the ABO blood groups (p=0.090) were similar between the two groups. Multiple logistic regression analyses (**Table 6**) showed that age (HR: 1.035, 95%CI: 1.019–1.052, p<0.001), Lauren classification (diffuse vs. intestinal: HR: 1.585, 95%CI: 1.048–2.397, p=0.029), Ki-67 index (HR: 1.017, 95%CI: 1.010–1.025, p=0.001), and EBV status (positive vs. negative: HR: 9.718, 95%CI: 3.495–27.021) were independent factors associated with the frequency of CPS≥10.

**Table 5:** Clinicopathological features associated with PD-L1 expression of CPS ≥ 10.

Variables	CPS < 10	CPS≥10	Statistic	p-value
Age(mean±SD)	56.1 ± 12.8	60.3 ± 11.3	-5.022	<0.001
BMI(mean±SD)	22.6 ± 3.0	22.2 ± 3.4	1.512	0.131
Sex[n(%)]			0.190	0.663
Male	397 (64.0)	203 (65.5)		
Female	223 (36.0)	107 (34.5)		
Diabetes[n(%)]			0.203	0.652
No	556 (89.7)	275 (88.7)		
Yes	64 (10.3)	35 (11.3)		
Location[n(%)]			4.982	0.083
Upper	123 (19.8)	76 (24.5)		
Middle	130 (21.0)	49 (15.8)		
Lower	367 (59.2)	185 (59.7)		
Histology[n(%)]			2.826	0.093
Sig	241 (38.9)	103 (33.2)		
Others	379 (61.1)	207 (66.8)		
Lauren classification[n(%)]			6.027	0.049
Intestinal	153 (29.9)	63 (25.6)		
Diffuse	298 (58.2)	138 (56.1)		
Mix	61 (11.9)	45 (18.3)		
Grade[n(%)]			5.507	0.138
G1	39 (6.3)	9 (2.9)		
G2	103 (16.6)	47 (15.2)		
G3	473 (76.3)	251 (81.0)		
G4	5 (0.8)	3 (1.0)		
Tumor size[n(%)]			13.671	<0.001
<2cm	459 (74.0)	193 (62.3)		
≥5cm	161 (26.0)	117 (37.7)		
T stage[n(%)]			-1.126	0.260
T1a	92 (14.8)	27 (8.7)		
T1b	56 (9.0)	29 (9.4)		
T2	61 (9.8)	30 (9.7)		
T3	149 (24.0)	90 (29.0)		
T4a	180 (29.0)	98 (31.6)		
T4b	82 (13.2)	36 (11.6)		
N stage[n(%)]			-0.393	0.694
N0	234 (37.7)	111 (35.8)		
N1	58 (9.4)	42 (13.5)		
N2	86 (13.9)	47 (15.2)		
N3	242 (39.0)	110 (35.5)		
Metastasis[n(%)]			1.737	0.188
No	468 (75.5)	246 (79.4)		
Yes	152 (24.5)	64 (20.6)		
Ki-67 (%), (mean±SD)	53.7 ± 23.9	64.7 ± 22.9	-6.281	<0.001
S-100[n(%)]			0.020	0.888
Negative	213 (41.8)	101 (41.2)		
Positive	297 (58.2)	144 (58.8)		
CD-31[n(%)]			6.687	0.010
Negative	412 (80.3)	177 (72.0)		
Positive	101 (19.7)	69 (28.0)		
D-240[n(%)]			1.644	0.071
Negative	368 (71.7)	162 (65.3)		
Positive	145 (28.3)	86 (34.7)		
EBV[n(%)]			40.127	<0.001
Negative	556 (98.8)	259 (89.3)		
Positive	7 (1.2)	31 (10.7)		
dMMR[n(%)]			5.879	0.015
No	581 (95.2)	279 (91.2)		
Yes	29 (4.8)	27 (8.8)		
HER2[n(%)]			-0.821	0.411
0	409 (66.0)	210 (67.7)		
+	128 (20.6)	69 (22.3)		
++	47 (7.6)	19 (6.1)		
+++	36 (5.8)	12 (3.9)		
WBC (x 10~9/L)(mean±SD)	6.0 ± 1.8	6.1 ± 2.2	-0.497	0.619
MONO(x 10~9/L)(mean±SD)	0.5 ± 0.2	0.6 ± 1.7	-0.231	0.022
EOS(x 10~8/L)(mean±SD)	1.7 ± 1.3	1.9 ± 1.8	-2.311	0.021
NEU(x 10~9/L)(mean±SD)	3.6 ± 1.6	3.7 ± 1.9	-1.177	0.240
LYM(x 10~9/L)(mean±SD)	1.8 ± 0.7	1.7 ± 0.7	3.030	0.001
NLR(mean±SD)	2.2 ± 1.5	2.5 ± 1.9	-2.846	0.005
PLT(mean±SD)	250.8 ± 82.1	257.7 ± 99.2	-1.234	0.217
ABO blood group[n(%)]			6.499	0.090
A	201 (32.8)	78 (25.2)		
B	132 (21.5)	76 (24.5)		
AB	48 (7.8)	32 (10.3)		
O	232 (37.8)	124 (40.0)		

CPS, combined positive score; BMI, body mass index; EGJ, esophagus-gastric junction; EBV, The Epstein-Barr virus; dMMR, mismatch repair deficiency; Her 2, Human epidermal growth factor receptor 2; WBC, white blood cell count; MONO, mononuclear cell count; EOS, eosinophilic granulocyte count; NEU, neutrophil count; LYM, lymphocyte count; NLR, neutrophil/lymphocyte ratio; PLT, the platelet count.

**Table 6:** The Multivariate Logistic Regression Analyses of the PD-L1 expression of CPS ≥ 10.

Variables	B	S.E.	P value	HR (95%CI)
Age	0.035	0.008	<0.001	1.035 (1.019-1.052)
Lauren classification (Diffuse/Intestinal)	0.460	0.211	0.029	1.585 (1.048-2.397)
Lauren classification (Mix/Intestinal)	0.538	0.280	0.055	1.713 (0.990-2.966)
Size(≥5cm/<2cm)	0.266	0.210	0.205	1.305 (0.865-1.969)
Ki67	0.017	0.004	0.001	1.017 (1.010-1.025)
CD31(+/-)	0.253	0.207	0.221	1.288 (0.859-1.932)
EBV(+/-)	2.274	0.522	<0.001	9.718 (3.495-27.021)
dMMR(+/-)	0.586	0.357	0.100	1.797 (0.793-3.616)
MONO	0.419	0.526	0.426	1.520 (0.543-4.258)
EOS	0.172	0.608	0.778	1.187 (0.360-3.913)
LYM	-0.008	0.017	0.209	0.992 (0.960-1.026)
NLR	0.005	0.112	0.965	1.005 (0.807-1.251)

CPS, combined positive score; EBV, The Epstein-Barr virus; dMMR, mismatch repair deficiency; MONO, mononuclear cell count; EOS, eosinophilic granulocyte count; LYM, lymphocyte count; NLR, neutrophil/lymphocyte ratio; B, regression coefficient; S.E.: standard error.

### Linear Regression Analysis of CPS With Clinicopathological Features

On the basis of the multivariate logistic regression analyses of CPS(≥1/≥5/≥10) with clinicopathological features, linear regression analysis was further performed to demonstrate the multivariate linear correlation of CPS with age, Lauren classification, Ki-67 index, and EBV status (**Table S3**). The multivariate linear regression analysis confirmed that CPS was linearly correlated with age (p<0.001), Lauren classification (p=0.002), Ki-67 index (p<0.001), and EBV status (p<0.001).

## Discussion

The immune regulatory PD-1/PD-L1 axis can induce inhibitory immune signaling within activated T cells, destroying their antitumor immune response, and thus is an immune checkpoint target for immunotherapy in many malignancies including GC (4, 7). The KEYNOTE-059 (4) and ATTRACTION-2 (19) trials confirmed the favorable efficacy and tolerability of anti-PD-1/PD-L1 therapies as third-line treatment for advanced GC. The CheckMate-649 trial (20) suggested the superiority of immune checkpoint inhibitors as first-line treatment. More encouragingly, we noted that durable response and long-term benefits could only be achieved by checkpoint inhibitors such as anti-PD-1 therapy rather than chemotherapy (21, 22). However, predictive biomarkers for the efficacy of anti-PD-1 therapy are lagging behind these clinical data. Currently, the primary indication for anti-PD-1 therapy in GC is the expression of PD-L1. However, since immune therapy progress rapidly in GC treatment in reccent year, most hospitals are not equipped to reliably test for PD-L1 expression in routine work. This phenomenon has limited the use of anti-PD-1 therapy in GC. Thus, we investigated whether PD-L1 expression in GC was associated with other clinicopathological features that are accessible in most hospitals.

PD-L1 protein expression is assessed in GC using CPS, which is the number of PD-L1 positively-stained cells (i.e., tumor cells, lymphocytes, and macrophages) divided by the total number of viable tumor cells, multiplied by 100%. A specimen is considered to have positive PD-L1 expression when CPS≥1. Consistently, Pembrolizumab is approved by the USA FDA for GC patients with positive PD-L1 expression (CPS≥1). The CheckMate-649 trial (20) presented survival benefits in patients with CPS≥5 following nivolumab treatment (HR: 0.71, 98.4%CI, 0.59–0.86, p<0.0001). The CheckMate-032 trial showed that PD-L1 expression by CPS demonstrated a stronger association with overall survival at higher cutoffs than PD-L1 expression on tumor cells (response rates of PD-L1 cutoff: <1 vs. ≥1 vs. ≥5 vs. ≥10: 0% vs. 28% vs. 41% vs. 55%) (11).While the KEYNOTE-062 trial which enrolled 763 patients with untreated, locally advanced/unresectable or metastatic GC with PD-L1 CPS ≥1, also analysed the efficiency of the subgroup of CPS≥10 (23). Meanwhile, many ongoing trials (e.g., NCT04139135 and NCT04744649) are currently exploring the effectiveness of neoadjuvant immunotherapy for GC using the cut-off of CPS≥10 to make sure there is a response to anti-PD-1 antibodies. However, all the CPS results regarding advanced GC and neoadjuvant treatment were tested on tissue from gastric mucosa biopsy. While for resectable GC, whether the CPS characteristics are different from the CheckMate-032 trial remains unknown. Our data answered this question and showed the significant CPS discrepancy between gastric mucosa biopsy and postoperative pathology. Our analysis also revealed that CPS≥1 was seen in 62.3% of patients (579/930), CPS≥5 was seen in 49.2% of patients (458/930), and CPS≥10 was seen in 33.3% of patients (310/930). While in CheckMate-032 trial, the percentages of CPS ≥1, ≥5, and ≥10 were 32%, 10%, and 8%, respectively. Another Chinese cohort analysis showed that 37.3% of cases (205/550) presented PD-L1 expression in tumor cells or tumor-infiltrating immune cells (24). Another Asian cohort study revealed that PD-L1 IHC scores were positive in 22.8% of patients (25). These results suggested that the spatial heterogeneity of PD-1 expression resulted in the assessments of gastric mucosa biopsy, such as those performed in previous studies, inaccurate in advanced GC. Accordingly, the potential beneficiaries of immune checkpoint inhibitors are likely broader than what has been reported. This phenomenon indicated that in clinical practice we should obtained tumor tissue from as many sites as possible when performing forceps biopsy to assess the PD-L1 expression.

Then, we searched for associations between PD-L1 expression and other clinicopathological features. The aim of this work was to identify new biomarkers associated with PD-L1 expression that are available in Chinese hospitals. Our analysis confirmed that CPS for PD-L1 was linearly correlated with age, Lauren classification, Ki-67 index, and EBV status. The Ki-67 index and Lauren classifications are both very accessible assays in most Chinese hospitals and might also be potential biomarkers for the efficacy of anti-PD-1 therapy. Thus, it is worth exploring their potential relationship with outcomes of anti-PD-1 therapy.

The multiplex immunofluorescence staining of non-small cell lung cancer (NSCLC) has revealed that Ki-67 index, along with cytokeratin, PD-L1, PD1, CD8, and CD68 are key components of the immune response to NSCLC (26). This finding was provided to assist others to apply similar methods to further understand the immune response to NSCLC. Zhao et al. (27) also showed that compared with those with negative PD-L1 expression, NSCLC patients with positive PD-L1 expression had significantly higher rates of lymph node metastasis (64.9% vs. 27.5%, p<0.01), more advanced tumor stage (p<0.01) and Ki-67 index (P<0.01), and thus concluded that positive PD-L1 expression was associated with more aggressive pathological features and poorer prognosis in advanced-stage NSCLC. Similarly, Pawelczyk et al. (28) also found that PD-L1 expression was associated with increased tumor proliferation and aggressiveness. In line with these NSCLC data, it was also found that PD-L1 expression was correlated with clinicopathologic parameters in breast cancer, including lymphovascular invasion and Ki-67 index (29). Accordantly, another study showed that PD-L1 expression was significantly associated with age and high Ki-67 index in breast cancer (30). Furthermore, some studies have even indicated that high Ki-67 index is a strong predictor of pathologic complete response in HER2+ breast cancer (31). Recently, it has been shown that positive Ki-67 and PD-L1 expression in post-neoadjuvant chemotherapy radical cystectomy samples was associated with inferior overall survival and the absence of tumor downstaging. IHC of Ki-67 and PD-L1 could help select patients for adjuvant therapy in post-neoadjuvant chemotherapy muscle-invasive bladder cancer (32). Wang et al. also demonstrated that high Ki-67 index was associated with a higher TNM stage and was an independent predictor of unfavorable prognosis in colorectal cancer (33). Thus, whether the Ki-67 index can improve the efficiency of predicting anti-PD-1 response in GC should be explored. Regarding Lauren classification, previous report has also demonstrated a significant association between PD-L1 status and Lauren classification (34). Histology pattern of Lauren classification included intestinal type, diffuse type and mixed type. The intestinal-type maintains the glandular appearance, which is concerned in an environmental factor; While the diffuse type shows diffusely infiltrating cells without glandular architecture which is used to be concerned in genetic factors. And the mixed type presents both intestinal type and diffuse type in the tumor specimen (35). Of coures, the Lauren classification is associated with biological behavior in GC (35); thus, finding ways to apply this information to identify tumor subsets and develop molecularly tailored, individualized immunotherapy benefits is goal for future studies.

EBV positivity has been proposed to be a predictive biomarker for anti-PD-1 response in GC patients (9, 10). Our analysis showed that EBV-positive GC is more prone to high PD-L1 expression. Consistently, previous studies have also shown that PD-L1 expression by tumor cells appears to be more common in EBV-positive GC (36). Another Asian cohort also demonstrated that PD-L1 expression was associated with distinct clinicopathological features, including dMMR and EBV positivity (25). Separate follow-up studies have also shown that EBV+ tumors exhibit robust PD-L1 expression both in cancer cells and immune cells (37). The mechanism might be that EBV-positive GCs are characterized by marked intra- or peri-tumoral immune cell infiltration and often exhibit genomic amplification of the chromosome 9 locus containing the genes encoding PD-L1 and PD-L2 (38). In 2018, Kim et al. provide insight into the molecular features associated with response to pembrolizumab in patients with metastatic GC and provided potentially relevant biomarkers for selecting patients who may derive greater benefit from PD-1 inhibition (10). Their results showed that dramatic responses to pembrolizumab were observed in patients with EBV-positive tumors (100% overall response rate in EBV-positive metastatic GC).

And we have noted that there is a synergetic effect on tumor reduction in some pre-clinical research when combining anti-HER2 and anti-PD-1 therapies (39). Moreover, the clinical benefit for the combination of anti-HER2 anti-PD-1 therapies, and chemotherapy for patients with HER2-positive GC have been revealed in clinical trial (40). On the basis of it, the phase III KEYNOTE-811 trial was conducted to investigate the efficacy whether pembrolizumab or placebo in coadministration with trastuzumab and the investigator`s choice of chemotherapy in participants with previously untreated unresectable or metastatic, HER2-positive gastric or gastro-oesophageal junction adenocarcinoma. And encouragingly, the results of KEYNOTE-811 trial suggested that adding pembrolizumab to trastuzumab and chemotherapy markedly enhanced the treatment efficacy (41).

These primary data indicate that HER-2 status is also the potential biomarker for anti-PD-1 therapy and encouraged us to investigate whether HER-2 status was correlated with PDL1 expression. Furthermore, another Chinese cohort suggested that PD-L1 expression was more common in HER2-negative tumors compared with HER-2-positive tumors (39.0% vs. 24.2%, P=0.020) (24). However, our data showed that PD-L1 expression was not associated with the HER-2 status in GC. Thus, the previous clinical synergetic effect of anti-HER-2 and anti-PD-1 may be attributed to other mechanisms. For example, preclinical study indicated that trastuzumab could upregulate expression of PD-L1 through engagement of immune effector cells may function as a potential mechanism (42). Also, it has been uncovered that combination of anti-HER-2 and anti-PD-1 therapies could improve HER-2-specific T cell responses, boost immune cell trafficking and weaken gathering of peripheral memory T cells (39, 43).

It is also noteworthy that our data showed that PD-L1 expression was not related to tumor stage. In contrast, many previous studies have indicated that PD-L1 expression is significantly associated with tumor stage. As early as 2014, Muenst et al. showed that PD-L1 expression was significantly associated with tumor size, AJCC primary tumor classification, tumor grade, and lymph node status in breast cancer (30). Similarly, Uhercik et al. (44) demonstrated significant transcript level reductions in PD-L1 in patients who developed metastases, as well as in those who had local recurrence compared with patients who remained disease-free. Consistently, PD-L1 expression in NSCLC was also increased in higher malignancy grades (p<0.001) and in higher lymph node status (p=0.043) (28). Furthermore, a systematic review and meta-analysis revealed that GC patients with deeper tumor infiltration, positive lymph node metastasis, and positive venous invasion were more likely to express PD-L1 (45). In contrast, our data showed that PD-L1 expression was not associated with the TNM stage in GC. Therefore, we propose that immunotherapy could also be explored for less advanced GC, rather than only for stage IV. However, until now, immunotherapy only been demonstrated in late-stage GC (4, 7). Whether locally advanced GC has enough PD-L1 expression to work with anti-PD-1/PD-L1 therapy remains unknown. Thus, our primary evidence provided perspective on this clinical question. Additionally, neoadjuvant chemotherapy or perioperative chemotherapy have been proposed to improve outcomes, which can increase the R0 resection rate by shrinking tumor size. Moreover, potential prognosis-related factors like micrometastases can be better addressed by chemotherapy prior to surgery (46, 47). Theoretically, neoadjuvant immunotherapy or perioperative immunotherapy for locally advanced GC could benefit patients. Therefore, the situation in which locally advanced GC has abundant PD-L1 expression similar to late-stage GC is supported by our data and provides a solid theoretical foundation for neoadjuvant immunotherapy or perioperative immunotherapy. Therefore, on the basis of our results showing locally advanced GC has a similar frequency of PD-L1 expression as late-stage GC, we designed a clinical trial to investigate the safety and effectivity of neoadjuvant immunotherapy for locally advanced GC (NCT04744649).

## Conclusions

PD-L1 expression showed strong intratumoral heterogeneity in GC; thus, in clinical practice, multiple biopsies should be recommended for accurate reflection of PD-L1 expression in GC. Age, Ki67 index, and Lauren classification, which is popular and accessible in most hospitals, should be further explored as potential biomarkers for anti-PD-1 therapy.

## Data Availability Statement

The raw data supporting the conclusions of this article will be made available by the authors, without undue reservation.

## Ethics Statement

The studies involving human participants were reviewed and approved by Ethics Committee of Nanfang Hospital, Southern Medical University. The patients/participants provided their written informed consent to participate in this study.

## Author Contributions

LZ, and JY made substantial contributions to the conception and design, and interpretation of data. XC, HZ, and MW contributed in drafting the manuscript or critically revising it for important intellectual content. HL, YH, TL, HC, MZ, and TC collected and analyzed the data. Each author participated sufficiently in the work to take public responsibility for appropriate portions of the content and agreed to be accountable for all aspects of the work in ensuring that questions related to the accuracy or integrity of any part of the work are appropriately investigated and resolved. All authors contributed to the article and approved the submitted version.

## Funding

This work was supported by grants from the Guangdong Provincial Key Laboratory of Precision Medicine for Gastrointestinal Cancer (2020B121201004), and National Natural Science Foundation of China (81902444) Science, Technology Program of Guangzhou (201903010072) and Climbing Program, Special Fund of Guangdong Province(No.pdjh2022a0093).

## Conflict of Interest

The authors declare that the paper was conducted in the absence of any commercial or financial relationships that could be construed as a potential conflict of interest.

## Publisher’s Note

All claims expressed in this article are solely those of the authors and do not necessarily represent those of their affiliated organizations, or those of the publisher, the editors and the reviewers. Any product that may be evaluated in this article, or claim that may be made by its manufacturer, is not guaranteed or endorsed by the publisher.
