# Corrigendum: Relationship between programmed death ligand 1 expression and other clinicopathological features in a large cohort of gastric cancer patients

**DOI:** 10.3389/fimmu.2023.1235273

**Published:** 2023-06-15

**Authors:** Xinhua Chen, Huimin Zhang, Minghao Wang, Hao Liu, Yanfeng Hu, Tian Lin, Hao Chen, Mingli Zhao, Tao Chen, Guoxin Li, Jiang Yu, Liying Zhao

**Affiliations:** ^1^ Department of General Surgery and Guangdong Provincial Key Laboratory of Precision Medicine for Gastrointestinal Tumor, Nanfang Hospital, The First School of Clinical Medicine, Southern Medical University, Guangzhou, China; ^2^ The First Clinical Medical School, Southern Medical University, Guangzhou, China

**Keywords:** gastric cancer, PD-L1, CPS, Ki-67, biopsy, pathology

In the published article, there was an error in the author list, and author Guoxin Li was listed erroneously, and should be denoted as a corresponding author. The corrected author list appears below.

Xinhua Chen^1†^, Huimin Zhang^2†^, Minghao Wang^1†^, Hao Liu^1^, Yanfeng Hu^1^, Tian Lin^1^, Hao Chen^1^, Mingli Zhao^1^, Tao Chen^1^, Guoxin Li^1*^, Jiang Yu^1*^, Liying Zhao^1*^

The authors apologize for this error and state that this does not change the scientific conclusions of the article in any way. The original article has been updated.

