# Supplementary material for: Relationship Between Programmed Death Ligand 1 Expression and Other Clinicopathological Features in a Large Cohort of Gastric Cancer Patients
Source: Front Immunol. 2022 Mar 25;13:783695. doi: 10.3389/fimmu.2022.783695 (PMC8990248; doi:10.3389/fimmu.2022.783695)
Supplement: Supplementary file 1 [file Table_1.doc]

| Table S1.Clinicopathological Features Associated with PD-L1 expression of CPS ≥ 5 | | | | |
| --- | --- | --- | --- | --- |
| Variables | CPS < 5 | CPS≥5 | Statistic | p-value |
| Age(mean±SD) | 56.1±12.7 | 59.0±12.0 | 3.636 | <0.001 |
| BMI(mean±SD) | 22.5±3.0 | 22.4±3.3 | -0.858 | 0.393 |
| Sex[n(%)] |  |  | 0.041 | 0.839 |
| Male | 306(64.8) | 294(64.2) |  |  |
| Female | 166(35.2) | 164(35.8) |  |  |
| Diabetes[n(%)] |  |  | 3.075 | 0.080 |
| No | 430(91.1) | 401(87.6) |  |  |
| Yes | 42(8.9) | 57(12.4) |  |  |
| Location[n(%)] |  |  | 2.500 | 0.287 |
| Upper | 96(20.3) | 103(22.5) |  |  |
| Middle | 100(21.2) | 79(17.2) |  |  |
| Lower | 276(58.5) | 276(60.3) |  |  |
| Histology[n(%)] |  |  | 0.037 | 0.848 |
| Sig | 176(37.3) | 168(36.7) |  |  |
| Others | 296(62.7) | 290(63.3) |  |  |
| Lauren classification[n(%)] |  |  | 6.809 | 0.033 |
| Intestinal | 121(31.5) | 95(25.4) |  |  |
| Diffuse | 220(57.3) | 216(57.8) |  |  |
| Mix | 43(11.2) | 63(16.8) |  |  |
| Grade[n(%)] |  |  | 5.989 | 0.112 |
| G1 | 32(6.8) | 16(3.5) |  |  |
| G2 | 80(16.9) | 70(15.3) |  |  |
| G3 | 356(75.4) | 368(80.3) |  |  |
| G4 | 4(0.8) | 4(0.9) |  |  |
| Tumor size[n(%)] |  |  | 4.077 | 0.043 |
| <5cm | 345(73.1) | 307(67.0) |  |  |
| ≥5cm | 127(26.9) | 151(33.0) |  |  |
| T stage[n(%)] |  |  | -0.088 | 0.930 |
| T1a | 68(14.4) | 51(11.1) |  |  |
| T1b | 41(8.7) | 44(9.6) |  |  |
| T2 | 45(9.5) | 46(10.0) |  |  |
| T3 | 117(24.8) | 122(26.6) |  |  |
| T4a | 133(28.2) | 145(31.7) |  |  |
| T4b | 68(14.4) | 50(10.9) |  |  |
| N stage[n(%)] |  |  | -0.017 | 0.987 |
| N0 | 179(37.9) | 166(36.2) |  |  |
| N1 | 49(10.4) | 51(11.1) |  |  |
| N2 | 61(12.9) | 72(15.7) |  |  |
| N3 | 183(38.8) | 169(36.9) |  |  |
| Metastasis[n(%)] |  |  | 3.121 | 0.077 |
| No | 351(74.4) | 363(79.3) |  |  |
| Yes | 121(25.6) | 95(20.7) |  |  |
| Ki-67(%)(mean±SD) | 53.6±24.3 | 61.3±23.3 | 4.594 | <0.001 |
| S-100[n(%)] |  |  | 3.617 | 0.298 |
| Negative | 168(43.4) | 146(39.7) |  |  |
| Positive | 219(56.6) | 222(60.3) |  |  |
| CD-31[n(%)] |  |  | 9.336 | 0.001 |
| Negative | 320(82.5) | 269(72.5) |  |  |
| Positive | 68(17.5) | 102(27.5) |  |  |
| D-240[n(%)] |  |  | 4.515 | 0.034 |
| Negative | 283(73.1) | 247(66.0) |  |  |
| Positive | 104(26.9) | 127(34.0) |  |  |
| EBV[n(%)] |  |  | 15.924 | <0.001 |
| Negative | 420(98.4) | 395(92.7) |  |  |
| Positive | 7(1.6) | 31(7.3) |  |  |
| dMMR[n(%)] |  |  | 1.451 | 0.228 |
| No | 440(94.8) | 420(93.1) |  |  |
| Yes | 24(5.2) | 31(6.9) |  |  |
| HER2[n(%)] |  |  | -0.853 | 0.394 |
| 0 | 323(68.4) | 296(64.6) |  |  |
| + | 87(18.4) | 110(24.0) |  |  |
| ++ | 35(7.4) | 31(6.8) |  |  |
| +++ | 27(5.7) | 21(4.6) |  |  |
| WBC  (x 10~9/L)(mean±SD) | 6.1±2.1 | 6.0±2.1 | -0.244 | 0.807 |
| MONO(x 10~9/L)(mean±SD) | 0.5±0.2 | 0.6±1.4 | 1.509 | 0.132 |
| EOS(x 10~9/L)(mean±SD) | 0.2±0.1 | 0.2±0.2 | 1.768 | 0.077 |
| NEU(x 10~9/L)(mean±SD) | 3.6±1.6 | 3.6±1.8 | 0.149 | 0.882 |
| LYM(x 10~9/L)(mean±SD) | 1.7±0.6 | 1.7±0.7 | -1.891 | 0.059 |
| NLR(mean±SD) | 2.3±1.6 | 2.4±1.7 | 1.206 | 0.228 |
| PLT(mean±SD) | 249.3±80.3 | 256.1±96.5 | 1.165 | 0.244 |
| ABO blood group[n(%)] |  |  | 1.835 | 0.607 |
| A | 149(31.8) | 130(28.6) |  |  |
| B | 101(21.6) | 107(23.5) |  |  |
| AB | 37(7.9) | 43(9.5) |  |  |
| O | 181(38.7) | 175(38.5) |  |  |
| CPS: combined positive score; BMI: body mass index; EGJ: esophagus-gastric junction; EBV: The Epstein-Barr virus; dMMR: mismatch repair deficiency; Her 2: Human epidermal growth factor receptor 2; WBC: white blood cell count; MONO: mononuclear cell count; EOS: eosinophilic granulocyte count; NEU: neutrophil count; LYM: lymphocyte count; NLR: neutrophil/lymphocyte ratio; PLT: the platelet count. | | | | |
