# Supplementary material for: Relationship Between Programmed Death Ligand 1 Expression and Other Clinicopathological Features in a Large Cohort of Gastric Cancer Patients
Source: Front Immunol. 2022 Mar 25;13:783695. doi: 10.3389/fimmu.2022.783695 (PMC8990248; doi:10.3389/fimmu.2022.783695)
Supplement: Supplementary file 2 [file Table_2.doc]

| Table S2. The Multivariate Logistic Regression Analyses of The PD-L1 Expression of CPS ≥ 5 | | | | |
| --- | --- | --- | --- | --- |
| Variables | B | S.E. | P value | HR(95%CI) |
| Age | 0.028 | 0.007 | <0.001 | 1.029(1.015-1.043) |
| Lauren classification (Diffuse/Intestinal) | 0.457 | 0.190 | 0.016 | 1.579(1.088-2.292) |
| Lauren classification (Mix/Intestinal) | 0.586 | 0.261 | 0.025 | 1.797(1.077-2.977) |
| Size(≥5cm/<2cm) | 0.141 | 0.195 | 0.468 | 1.152(0.786-1.688) |
| Ki-67 | 0.011 | 0.002 | 0.002 | 1.011(1.004-1.018) |
| CD31(+/-) | 0.354 | 0.216 | 0.101 | 1.425(0.933-2.177) |
| D240(+/-) | 0.100 | 0.194 | 0.605 | 1.105(0.756-1.617) |
| EBV(+/-) | 1.490 | 0.514 | 0.004 | 4.439(1.620-12.160) |
| CPS: combined positive score; EBV: The Epstein-Barr virus; B: regression coefficient; S.E.: standard error. | | | | |
