# Supplementary material for: Relationship Between Programmed Death Ligand 1 Expression and Other Clinicopathological Features in a Large Cohort of Gastric Cancer Patients
Source: Front Immunol. 2022 Mar 25;13:783695. doi: 10.3389/fimmu.2022.783695 (PMC8990248; doi:10.3389/fimmu.2022.783695)
Supplement: Supplementary file 3 [file Table_3.doc]

| Table S3. Multivariate Linear Regression Analysis of CPS with Clinicopathological Features | | | |
| --- | --- | --- | --- |
| Variables | Correlation coefficient | | p-value |
| Age | 0.124 | | <0.001 |
| Lauren classification | 0.114 | | 0.002 |
| Ki67 | 0.220 | | <0.001 |
| EBV | 0.209 |  | <0.001 |
| CPS: combined positive score; EBV: The Epstein-Barr virus | | | |
